# Diagnostic Accuracy of Indocyanine Green Clearance Test for Different Stages of Liver Fibrosis and Cirrhosis

**DOI:** 10.3390/diagnostics13162663

**Published:** 2023-08-12

**Authors:** Lukas Luerken, Marco Dollinger, Andrea Goetz, Kirstin Utpatel, Michael Christian Doppler, Jakob Benedikt Weiss, Wibke Uller, André Ignee, Niklas Verloh, Michael Haimerl

**Affiliations:** 1 Department of Radiology, University Hospital Regensburg, 93053 Regensburg, Germany; lukas.luerken@ukr.de (L.L.);; 2 Department of Pathology, University Hospital Regensburg, 93053 Regensburg, Germany; 3 Department of Diagnostic and Interventional Radiology, Medical Center University of Freiburg, Faculty of Medicine, University of Freiburg, 79085 Freiburg, Germany; 4 Department of Gastroenterology, Hospital Wuerzburg Mitte, 97074 Wuerzburg, Germany; 5 Department of Diagnostic and Interventional Radiology, Hospital Wuerzburg Mitte, 97074 Wuerzburg, Germany

**Keywords:** hepatology, chronic liver disease, liver function, liver fibrosis, liver cirrhosis, diagnostic tests

## Abstract

(1) Background: This study aimed to correlate the indocyanine green clearance (ICG) test with histopathological grades of liver fibrosis and liver cirrhosis to assess its diagnostic accuracy in differentiating normal liver parenchyma from liver fibrosis and liver cirrhosis. (2) Methods: A total of 82 patients who received a histopathological liver examination, imaging, and ICG test within three months were included in this retrospective study. The histopathological level of fibrosis was graded using the Ishak scoring system, and the patients were divided into five categories: no liver fibrosis (NLF), mild liver fibrosis (MLF), advanced liver fibrosis (ALF), severe liver fibrosis (SLF), and liver cirrhosis (LC). The non-parametric Kruskal–Wallis test with post hoc pairwise comparison utilizing Mann–Whitney U tests and Bonferroni adjustment was used to analyze differences in the ICG test results between the patient groups. Cross correlation between the individual fibrosis/cirrhosis stages and the score of the ICG test was performed, and the sensitivity, specificity, and positive and negative predictive values were calculated for each model predicting liver fibrosis/cirrhosis. (3) Results: A significant difference (*p* ≤ 0.001) between stages of NLF, LF, and LC was found for the ICG parameters (ICG plasma disappearance rate (ICG-PDR) and ICG retention percentage at 15 min (ICG-R15)). The post hoc analysis revealed that NLF significantly differed from SLF (ICG-PDR: *p* = 0.001; ICG-R15: *p* = 0.001) and LC (ICG-PDR: *p* = 0.001; ICG-R15: *p* = 0.001). ALF also significantly differed from SLF (ICG-PDR: *p* = 0.033; ICG-R15: *p* = 0.034) and LC (ICG-PDR: *p* = 0.014; ICG-R15: *p* = 0.014). The sensitivity for detection of an initial stage of liver fibrosis compared to no liver fibrosis (Ishak  ≥  1) was 0.40; the corresponding specificity was 0.80. The differentiation of advanced liver fibrosis or cirrhosis (Ishak ≥ 4) compared to other stages of liver fibrosis was 0.75, with a specificity of 0.81. (4) Conclusions: This study shows that the ICG test, as a non-invasive diagnostic test, is able to differentiate patients with no liver fibrosis from patients with advanced liver fibrosis and liver cirrhosis. The ICG test seems to be helpful in monitoring patients with liver fibrosis regarding compensation levels, thus potentially enabling physicians to both detect progression from compensated liver fibrosis to advanced liver fibrosis and cirrhosis and to initiate antifibrotic treatment at an earlier stage.

## 1. Introduction

In the Western Hemisphere, chronic liver disease and liver cirrhosis are among the leading causes of death. Contributing to this growing problem is the epidemic increase in obesity, nonalcoholic fatty liver disease, and alcohol-related cirrhosis, as morbidity and mortality are directly correlated with the progression of liver fibrosis. In association with progressive liver damage, an accumulation of extracellular matrix proteins occurs, which may distort the hepatic architecture. The subsequent development of nodules of regenerating hepatocytes, fibrotic scars, and destructive liver architecture defines liver cirrhosis and is associated with various sequelae, such as portal hypertension and its clinical consequences or the risk for development of hepatocellular carcinoma. The increasing promise of antiviral therapies in slowing down or reversing fibrogenic progression of chronic liver disease has provided important targets for antifibrotic medications. Therefore, both continuous monitoring and early detection of liver fibrosis have significant therapeutic implications [1,2,3,4,5].

The evaluation of liver volume, clinical grading systems, and biochemical liver parameters is essential for the prognosis and clinical management of patients with chronic liver disease or who undergo liver surgery. In clinical routine, both static and dynamic liver function tests, as well as clinical scoring systems, are used to assess the prognosis of liver disease. In Europe, the Child–Pugh score and the model for the end-stage liver disease score (MELD score) are widely used to assess liver dysfunction. However, in clinical routine, obtaining a liver biopsy is still the gold standard in diagnosing and evaluating liver fibrosis (LF) and liver cirrhosis (LC). Liver biopsies may be prone to sampling errors and interobserver variability; are associated with risks of various complications, such as bleeding and infection; and are therefore poorly accepted by patients [6,7,8,9]. For histopathological examination, the Ishak score, also known as the modified Knodell or modified histology activity index (HAI) score, is used. It assesses the level of fibrosis, with scores ranging from 0 (no fibrosis) to 6 (cirrhosis).

A well-established gold standard for the quantitative assessment of global liver function is the indocyanine green (ICG) clearance test [10], which is routinely used to assess the functional liver reserve before liver resections. A strong correlation between the MELD score, the ^13^C-Methacetin breath test, and the CTP score has been observed [11,12,13].

After intravenous injection, ICG is removed from circulation exclusively by the liver [14]. After administration, ICG uptake into the hepatocytes is mainly mediated by the organic anion-transporting polypeptides (OATP1), which are located at the basolateral membrane of hepatocytes [15]. The cellular expression of OATP1 and consecutive hepatic uptake of ICG is dependent on hepatocyte function and the hepatocyte’s integrity. Liver fibrosis in particular and liver diseases in general are associated with reduced liver function. This leads to a downregulation of the expressed OATP1 transporters, resulting in reduced uptake of ICG into the hepatocytes. In LF and LC, downregulation or lower expression of organic anion-transporting polypeptides in hepatocytes initially were described in animal models [16,17,18].

This study aimed to correlate the indocyanine green clearance test with histopathological grades of LF and LC and to assess the diagnostic accuracy to differentiate normal liver parenchyma (NLF) from LF and LC.

## 2. Materials and Methods

### 2.1. Patients

Approval from the local institutional review and ethics board of the University Hospital Regensburg was obtained, and this retrospective study was performed in accordance with the relevant guidelines and regulations. Written informed consent was waived for this retrospective evaluation.

Data sources included electronic patient charts, the radiology information system, and a picture-archiving computer system. The inclusion criteria were histopathological liver examination, imaging, and an ICG test within three months. Exclusion criteria were insufficient liver samples with tissue lengths shorter than 15 mm or less than ten visible portal tracts and sampling errors.

### 2.2. ICG Test

To evaluate the ICG retention rate at 15 min (ICG-R15) and the ICG plasma disappearance rate (PDR) non-invasively, a LiMON pulse-densitometric system (Impulse Medical System, Munich, Bavaria, Germany) was applied. An amount of 0.5 mg per kilogram body weight of ICG (ICGPulsion, Munich, Bavaria, Germany) was injected intravenously as a bolus; thereafter, 10 mL of normal saline was flushed through the catheter. Intravenously administered ICG was noticed from fractional pulsatile changes in visual absorption. ICGR-15 and ICG-PDR were specified as elimination measurements by monoexponential transformation of the initial ICG concentration curve and backward extrapolation at time zero (100%); here, the decay is expressed as a percentage change over time. A PDR <17%/min was defined as a cutoff for liver dysfunction, as described by Haegele et al. [19].

### 2.3. Histopathological Examination

All samples were prepared with formalin and embedded in paraffin. Samples were stained with hematoxylin–eosin (HE) and Elastica van Gieson (EVG) according to standard protocols. EVG staining was used to evaluate LF. Collagen stained red, and the hepatocytes stained yellow.

An automatic needle device was used to obtain liver samples for the liver biopsies. The length of each biopsy specimen was measured, and the number of portal spaces was assessed. Exclusion criteria were tissue length below 15 mm and fewer than ten visible portal tracts. Only non-tumorous liver biopsies were included in this study. Biopsies or resection specimens were reviewed by a specialist in liver histopathology, who was blinded to the patient data. Fibrosis was graded using the Ishak scoring system [20].

### 2.4. Volumetric Analysis

Image segmentation was performed using the semiautomatic segmentation algorithm based on active contour (also known as “SNAKE”) with manual edge correction, as implemented by ITK-SNAP open-source software (version 3.8.0) [21].

### 2.5. Statistical Analysis

All statistical analyses were performed with IBM SPSS Statistics (Version 29, Chicago, IL, USA). Two-sided tests were used for all statistical calculations, with a statistical significance set at a level of 0.05. All data are presented as mean ± standard deviation. In order to investigate discrepancies among patient groups, the non-parametric Kruskal–Wallis test with post hoc pairwise comparison utilizing Bonferroni adjustment and Mann–Whitney U tests (Dunn-Bonferroni-Tests) was applied. Patient-group-related differences were given, as stratified by the Ishak score. Cross correlation between the individual fibrosis/cirrhosis stages and the score of the ICG test was performed, and the sensitivity, specificity, and positive and negative predictive values were calculated for each model predicting liver fibrosis/cirrhosis.

## 3. Results

### 3.1. Patients

One hundred and nineteen adult patients were initially included in the study between June 2012 and January 2020. Thirty-seven patients were excluded from this study due to insufficient liver samples (*n* = 19) or sampling errors (*n* = 18). Ultimately, 82 patients (52 men and 32 women; mean age: 60.2 ± 15 years) were included in our study. Patients had liver resections due to suspected focal hepatic lesions (*n* = 76) or liver biopsies to monitor active hepatocellular carcinomas in cases of known LC (*n* = 6). The mean height of the patients was 172.2 ± 9.6 cm, the mean weight was 76.1 ± 17.2 kg, and the mean body mass index (BMI) was 25.5 ± 4.4.

The patients were subdivided into the following five categories: NLF (no liver fibrosis = Ishak 0; *n* = 29), MLF (mild liver fibrosis = Ishak 1; *n* = 16), ALF (advanced liver fibrosis = Ishak 2 + 3; *n* = 17), SLF (severe liver fibrosis = Ishak 4 + 5; *n* = 13), and LC (liver cirrhosis = Ishak 6; *n* = 7).

The non-parametric Kruskal–Wallis test showed no significant differences between the patient groups for patient age, gender, body weight, height, or BMI. Table 1 provides a summary of the patient characteristics. Only the gender distribution showed a tendency toward a male predilection for the patient groups with different stages of liver fibrosis and liver cirrhosis (H = 7.090 (df = 4), *p* = 0.131).

### 3.2. Correlation of ICG Results and Liver Volume with Ishak Scores

**Figure 1:**
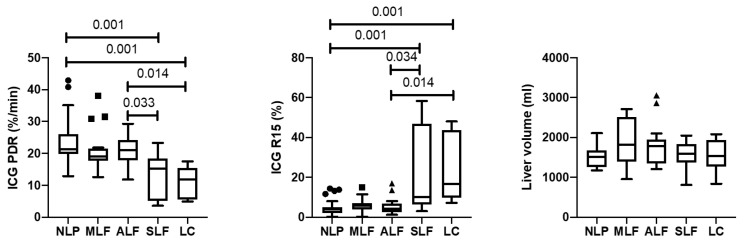
ICG results and liver volume of the patients classified according to the degree of specific fibrosis/cirrhosis.

A significant difference (*p* ≤ 0.001) between stages of NLF, LF, and LC was found for the ICG parameters (ICG-PDR and ICG-R15). No significant difference was found in liver volume. The post hoc analysis (Figure 1) revealed that NLF (ICG-PDR, 22.8 ± 7.6; ICG-R15, 4.7 ± 4.0) significantly differed from SLF (ICG-PDR, 13.1 ± 7.1, *p* = 0.001; ICG-R15, 22.5 ± 21.2, *p* = 0.001) and LC (ICG-PDR, 11.3 ± 4.9, *p* = 0.001; ICG-R15, 23.0 ± 17.0, *p* = 0.001). No significant differences were found between NLF, MLF, and ALF for ICG-PDR and ICG-R15; however, ALF significantly differed from SLF (ICG-PDR, *p* = 0.033; *p* = 0.034) and LC (ICG-PDR, *p* = 0.014; ICG-R15, *p* = 0.014). No significant differences were observed between SLF and LC for ICG-PDR and ICG-R15.

The non-parametric Kruskal–Wallis test with post hoc pairwise comparison using Mann–Whitney U tests and Bonferroni adjustment (Dunn-Bonferroni-Tests) was used to compare the following groups: NLF, no liver fibrosis; MLF, mild liver fibrosis; ALF, advanced liver fibrosis; SLF, severe liver fibrosis; and LC, liver cirrhosis. Table 2 provides an overview over the ICG test results of the different patient groups.

### 3.3. Diagnostic Accuracy of ICG in Differentiating Stages of Liver Fibrosis/Cirrhosis

The described cutoff value of ICG (PDR < 17%/min) indicating impaired liver function was applied to this data sample. Table 3 illustrates consequent specificities and sensitivities, as well as related predictive values (positive and negative). The sensitivity for detection of the initial liver fibrosis stage compared to the stage with no liver fibrosis (Ishak ≥ 1) was 0.40; the related specificity was 0.80. The differentiation of advanced liver fibrosis or cirrhosis (Ishak ≥ 4) compared to other stages of liver fibrosis was 0.75, with a specificity of 0.81.

## 4. Discussion

The non-invasive assessment of liver cirrhosis and the extent of liver disease as an expression of liver function is of high clinical relevance. In this study, the use of ICG to assess the degree of liver fibrosis, expressed as the histological Ishak score, was examined to evaluate the diagnostic accuracy of staging different liver fibrosis/cirrhosis levels. Determination of liver function plays a critical role in monitoring patients with liver dysfunction, selecting therapeutic approaches for patients with malignant liver disease, and optimizing patient selection to avoid post hepatectomy liver failure after liver resection. Although the causes of liver failure are multifactorial and depend on the patient’s comorbidities or pre-existing liver disease, inadequate postoperative residual liver function is one of the major factors for postoperative mortality after liver resection and has become an important issue. Various scoring systems (e.g., MELD score and CTP score) and various static tests, such as measurement of liver enzymes, protein synthesis (albumin and coagulation factors), and bilirubin excretion can be used in clinical practice to assess global liver function. In addition to the ICG test, which was used in this study, the dynamic ^13^C-methacetin breath test (LiMAx) can be used as a metabolic liver function test for evaluation of global liver function. The ^13^C-methacetin breath test and has also proven to be a reliable diagnostic tool in transplantation, liver resection, and monitoring of septic patients. Intravenously administered ^13^C-methacetin is metabolized by hepatocyte-specific enzyme cytochrome P450 1A2 (CYP1A2) to paracetamol and non-radioactive ^13^CO_2_. The exhaled ^13^CO_2_ leads to a change in the normal breath ^13^CO_2_/^12^CO_2_ ratio, which is determined by a specific analyzer based on modified non-dispersive isotope-selective infrared spectroscopy. The resulting change in the ^13^CO_2_/^12^CO_2_ ratio measured by breath analysis is then used to calculate liver maximal capacity (LiMAx test), which reports liver function. According to Stockmann et al., the LiMAx value for patients with reduced liver function was defined as <315 μg/kg/h in tests in healthy volunteers. However, the LiMAx test requires a high level of patient compliance and, in contrast to the ICG test, is very time-consuming and expensive [22].

In addition to the ICG and LiMAx tests, there are other non-invasive methods for determining liver function, such as tests using transient elastography like FibroScan and various scoring systems based on laboratory values. However, no studies are known to us that compare the multiple tests in terms of their significance in assessing liver function in patients with liver fibrosis or liver cirrhosis. A prospective study by Blüthner et al. including 22 patients with intestinal failure receiving total parenteral nutrition suggests that the LiMAx test may have a higher sensitivity in detecting early changes in liver function compared to the ICG test, FibroScan, and laboratory tests [23]. Whether this translates to patients with liver fibrosis and cirrhosis is unclear requires further, larger-scale studies.

It is well known that the ICG clearance test is a sensitive indicator for evaluating liver function [24] and is well established in screening patients before liver surgery [19] and assessing graft and donor function in liver transplantation [25]. Other indications for using ICG clearance are the assessment of liver function in critically ill patients [26] and patients with acute liver failure [27]. Only a few studies have investigated the ICG test for the assessment of liver cirrhosis and linked it to clinical scores [11,12,13,28]. To the best of our knowledge, there no study has been published regarding the ICG clearance test in correlation to the histopathological Ishak score.

In our study, the mean ICG-R15 was 4.9 for NLF, 10.8 for LF, and 23.0 for LC, with a significant difference. These results are in line with previously reported results on patients with CTP results (CTP-a, 7.3%; CTP-B, 13.7%) [12]. Although ICG could not differentiate between NLF, MLF, and ALF, there is a significant difference if NLF is compared to SLF and LC, indicating that patients with initial liver fibrosis still have a compensated liver function. ICG with a cutoff of PDR 17%/min for the initial liver fibrosis showed a high positive predictive value of 78%; however, the negative predictive value was poor, at only 42% (sensitivity 40%, specificity 79%). The ICG test seems to be very suitable for staging a high degree of liver fibrosis (Ishak ≥ 4), with a negative predictive value of 91% (sensitivity, 75%; specificity, 81%).

Notably, 44.7% of the patients in this study suffered from liver metastasis and had prior chemotherapy before liver resection. As neoadjuvant therapy is known to induce sinusoidal obstruction syndrome and steatohepatitis, preoperative liver function testing is crucial in these patients and may interfere with ICG clarification [29,30]. However, no significant difference was found between patients with and without prior chemotherapy.

Our study is subject to several limitations. First, the number of included patients was small. A stronger correlation between ICG and liver fibrosis score could possibly have been obtained if our study had included a larger number of patients in a multicenter setup. In particular, the number of patients with Ishak scores of 3 and 5 was low; however, patients with Ishak scores of 2 and 3, as well as Ishak scores of 4 and 5, were grouped together to form an advanced liver fibrosis group and a severe liver fibrosis group, respectively. A larger patient cohort and a prospective design would improve the statistical power of our analysis. Second, only one experienced pathologist performed histopathological examination, which could have led to reduced reliability of the histopathological results. Third, for the histopathological examinations, both resection specimens and an automatic needle device were used to obtain the liver samples. This may be a source of variation due to the potential inhomogeneous distribution of parenchymal changes throughout the liver parenchyma.

## 5. Conclusions

In conclusion, this study shows that the ICG test, as a non-invasive diagnostic test, is able to differentiate patients with no liver fibrosis from patients with advanced liver fibrosis and liver cirrhosis. There is a significant difference of ICG clearance compared to SLF and LC, indicating that patients with initial liver fibrosis still have a compensated liver function. The ICG test seems to be helpful in monitoring patients with LF regarding compensation levels, potentially enabling physicians to both detect progression from compensated liver fibrosis to advanced liver fibrosis and cirrhosis and to initiate antifibrotic treatment at an earlier stage.

## Figures and Tables

**Table 1:** Patient characteristics.

	All (*n* = 82)	NLF (*n* = 29)	MLF (*n* = 16)	ALF (*n* = 17)	SLF (*n* = 13)	LC (*n* = 7)	H (df), *p* Value *
Age (years)	60.2 ± 15.1	59.6 ± 15.8	61.3 ± 17.1	59.8 ± 14.3	60.8 ± 14.9	60.1 ± 13.0	0.384 (4),
							0.984
Male	50 (61%)	13 (45%)	9 (56%)	12 (71%)	10 (77%)	6 (86%)	7.090 (4),
(%)							0.131
Height (cm)	172.2 ± 9.6	171.6 ± 8.9	170.5 ± 9.6	173.0 ± 10.3	173.0 ± 11.8	173.7 ± 8.4	0.924 (4),
							0.921
Weight (kg)	76.1 ± 17.2	73.5 ± 17.5	72.9 ± 16.1	80.5 ± 18.3	75.5 ± 15.3	84.7 ± 15.7	3.392 (4),
							0.494
BMI (kg/m^2^)	25.5 ± 4.4	24.7 ± 4.2	24.9 ± 4.2	26.4 ± 4.5	25.2 ± 4.1	28.1 ± 5.4	2.624 (4),
							0.623

The values indicate the mean ± the standard deviation unless otherwise indicated. NLF = no liver fibrosis; MLF = mild liver fibrosis; ALF = advanced liver fibrosis; SLF = severe liver fibrosis; LC = liver cirrhosis; BMI = body mass index. *: non-parametric Kruskal–Wallis test.

**Table 2:** ICG results and liver volume of the patients classified according to the degree of specific fibrosis/cirrhosis.

Ishak		*n*	ICG-PDR	ICG-R15	Liver Volume (mL)
0	NLF	29	22.8 ± 7.6	4.7 ± 4.0	1539 ± 268
1	MLF	16	21.0 ± 6.7	5.9 ± 3.8	1859 ± 565
2	ALF	13	20.8 ± 4.6	5.5 ± 4.2	1801 ± 520
3		4			
4	SLF	9	13.1 ± 7.1	22.5 ± 21.2	1547 ± 337
5		4			
6	LC	7	11.3 ± 4.9	23.0 ± 17.0	1533 ± 415

The values indicate the mean ± the standard deviation. NLF = no liver fibrosis; MLF = mild liver fibrosis; ALF = advanced liver fibrosis; SLF = severe liver fibrosis; LC = liver cirrhosis.

**Table 3:** Diagnostic accuracy of ICG (cutoff < 17%/min).

	Sensitivity	Specificity	PPV	NPV
Ishak ≥ 1	0.40	0.79	0.78	0.42
Ishak ≥ 2	0.49	0.80	0.67	0.67
Ishak ≥ 4	0.75	0.81	0.56	0.91
Ishak = 6	0.86	0.72	0.22	0.98

The sensitivity and specificity and the corresponding positive (PPV) and negative predictive values (NPV) of the cutoff value < 17%/min are shown for MLF or greater (Ishak  ≥  1), ALF or greater (Ishak  ≥  2), SLF or greater (Ishak  ≥  4), and LC (Ishak  =  6).

## Data Availability

The source data presented in this study are available upon request from the corresponding author. The source data are not publicly available due to patient privacy.
